# Walnut (*Juglans regia* L.) shell pyroligneous acid: chemical constituents and functional applications

**DOI:** 10.1039/c8ra03684e

**Published:** 2018-06-19

**Authors:** Ali Jahanban-Esfahlan, Ryszard Amarowicz

**Affiliations:** Student Research Committee, Tabriz University of Medical Sciences Tabriz Iran a,jahanban@gmail.com +98 41 33366581 +98 41 33366581; Infectious and Tropical Diseases Research Center, Tabriz University of Medical Sciences Tabriz Iran; Nutrition Research Center, Tabriz University of Medical Sciences Tabriz Iran; Division of Food Sciences, Institute of Animal Reproduction and Food Research of the Polish Academy of Sciences Olsztyn Poland

## Abstract

Upon the processing of different agricultural products, considerable amounts of by-products and bio-wastes are produced and discarded or burnt as fuel, which are a potential source of valuable compounds. Over the past several decades, plant by-products have been recognized as a source of nutraceutical components, including dietary fibers, phenolics, and many other useful compounds. The walnut is known as an important tree nut. The shell of a walnut is the middle part of the fruit and it is a waste product of walnut processing industries. Recently, pyroligneous acids from the walnut shell have been receiving much-increasing interest because of their excellent antimicrobial and antioxidant activities. Hence, this review deals with the recent scientific literature on walnut shell pyroligneous acids and their chemical composition as well as their functional applications.

## Abstract 2

Upon the processing of different agricultural products, considerable amounts of by-products and bio-wastes are produced and discarded or burnt as fuel, which are a potential source of valuable compounds.

## Graphical abstract

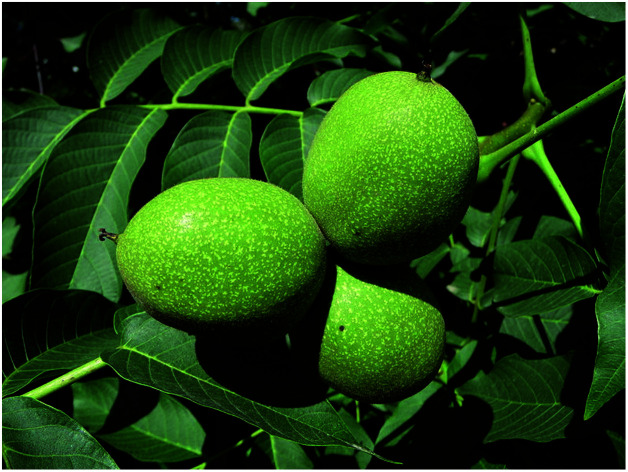

## 1. Introduction

Fruit production is an important branch of horticulture. Fruit plays a key part in keeping everyone fit and healthy, because it provides people with the healthy nutrients needed to help the body function.^1,2^ In the last two decades, there has been an explosive interest in the utilization of plants waste materials, more generally known as “agricultural by-products”. Fruit shells and other agricultural wastes are potentially important sources for producing valuable compounds.^3^ These plant-based by-products are produced in high amounts and crop wastes are rich in different nutritional components with various uses.^4,5^ Recently, the utilization of by-products has been increased by food and pharmaceutical manufacturers for the production of valuable compounds from such cheap resources. Walnuts are one of the most important agricultural products, owing to their different uses within the food industry.^6^ Walnut shells have great potential because of their production in large amounts. In the walnut processing industry, upon separation of the kernel from the outer parts of the fruit, large amounts of husk and shell are produced. These materials are the major part (more than 60%) of the walnut fruit and are discarded or burnt as fuel without any useful applications. Unfortunately, this waste material is generally combusted directly *in situ* for heating purposes, whereas it could potentially be utilized both for the production of high-value-added chemicals and for various forms of fuel by means of a suitable process such as pyrolysis.^3,7^

In recent years, much attention has been paid to natural antioxidants originating from different parts of plant materials.^8,9^ There is public concern about conventional synthetic food additive antioxidants, such as butyl hydroxyanisole (BHA), dibutyl hydroxyl toluene (BHT), and propyl gallate, and, thus, there has been a tendency to use natural antioxidants.^10^ Many investigations have shown that the antioxidants, mainly phenolic compounds, present in plants could be advantageous for human wellbeing.^11^ Free radicals cause damage to different bio-macromolecules, such as DNA and proteins of the cells in the body, and subsequently cause the onset of some diseases such as cancers and coronary artery disease.^12–14^ The high radical scavenging capacity of antioxidants prevents the deleterious effects of free radicals in the human body.^14–18^ Recently, the antioxidant and antimicrobial properties of pyroligneous acids from walnut shells have been reported. This review describes recent scientific knowledge regarding the chemical composition and constituents of walnut shell pyroligneous acids as well as their functional applications.

## 2. Walnuts

The walnut is an important tree nut and it has been widely used in human nutrition since ancient times.^19^ It is known to be one of the most important non-timber forest products.^6^ The walnut tree belongs to the *Juglandaceae* family.^20^ It is the most widespread nut-bearing tree and is cultured in some parts of the world due to its valuable kernels.^21^ The walnut tree is native to central Asia, the western Himalayan chain and Kyrgyzstan, and it was cultivated in Europe as early as 1000 BC. Since then, it has spread and become well adapted to many regions with Mediterranean-type ecosystems throughout the World.^22^ The nutritional importance of the walnut is related to its seed or kernel.^23^ It is a nutrient-dense food, mainly owing to its oil content (up to 740 g kg^−1^ in some commercial varieties), which can be extracted easily by screw pressing and consumed without refining.^24,25^ The kernel of the walnut contains high amounts of antioxidants and other beneficial compounds.^19,26–29^ Recently, some investigations have focused on the anti-cancer effects of juglone (5-hydroxy-1,4-naphthoquinone). It is a phenolic compound with allelopathic activities, belonging to the class of naphthoquinones, and is synthesized in different parts of the fruit, bark, leaves, and roots of the walnut tree.^30–34^ It has been reported that the regular consumption of walnuts by humans can reduce the risk of cancer and coronary artery heart diseases.^13,17,35–42^ It has been reported that there are high levels of phenolic compounds in the seed coat (skin) of the seed, which are found to possess strong antioxidant properties.^22,28^

## 3. Walnut fruit

From the nutritional point of view, the fruit of the walnut is the main and most important part of the tree because it contains the seed. The fruit of the walnut consists of four distinct parts. The husk or hull is the name of the outer layer which has a green color. It cracks when the fruit is fully ripened. The shell is the name of the middle part of the walnut fruit and, after separation from the fruit husk, it is named the nut. The leathery skin is the third part of the walnut fruit and it surrounds the kernel. This thin layer protects the kernel of the walnut from different dangerous environmental factors, such as microbial contamination and ultraviolet irradiation.

## 4. Walnut fruit by-products

The walnut is characterized by its high nutritional value; although the scientific literature reported so far principally concerns the edible kernel.^19,43^ Even though the nutritional and commercial relevance of the walnut has to date been limited to its kernel, growing attention has been paid to the other parts of this fruit, including its skin, shell, and husk, and even the leaves or branches of the tree.^44–51^ Different properties of walnut fruit by-products, as valuable sources of bioactive compounds, have not been investigated comprehensively, and this causes the continuation of traditional uses for walnut waste products that nowadays are mostly discarded as waste material or burnt for heating applications.^20^ This objective has encouraged scientists to investigate the biological activity and functional properties of walnut fruit waste products in order to make better use of them. In this sense, the scientific literature about the physicochemical and phytochemical composition of walnut meat along with its related co-products recommends new applications and promising uses, such as functional ingredients, value-added foods, and feeds; as well as a source of bioactive phytochemicals to be included in the cosmetic, food, and pharmaceutical industries. These efforts have provided interesting information regarding the high capacity of the bioactive compounds of walnut waste products in the prevention of degenerative diseases in relation to oxidative stress and inflammation in the human body.^36,45,52–58^

## 5. Walnut shell

A great amount of walnut shell is available after fruit processing in walnut kernel production centers, yet only a limited amount has been used in industry.^59^ In fact, most of it is discarded as waste material or used as fuel for burning applications.^44^ But this causes pollution of the environment, and it also has a low utility value. Therefore, it is necessary to find other, better uses for walnut shells. Walnut shell composition is very similar to that of other wood biomass because cellulose, hemicellulose, and lignin are the main components.^60–66^

Various methods have been suggested for the hydrolysis of cellulose and hemicellulose in wood biomass as a means of producing carbohydrates, which are good sources for the production of bioethanol.^59^ Another effective method for the utilization of walnut shells is the production of charcoal and activated carbon by executing pyrolysis.^67^ During the production of charcoal, the generated gases are usually discharged into the air. If they could be condensed and used, it would significantly increase the value of walnut shells. Currently, studies of walnut shell pyrolysis have mainly focused on the process conditions, the kinetics and charcoal properties. Aygün, *et al.*^68^ developed a method for the preparation of activated carbon from bio-resources, including walnut shells. The research focused on the influences of pyrolytic conditions on the yield of hydrogen gas, such as the temperature and the amount of added catalyst. Demirbas^7^ considered the process of slow pyrolysis of the stones of four types of nuts, including the walnut, with an emphasis on the influence of temperature on the yield of pyrolytic products, such as carbon, liquid (mainly viscous oil), and gas. Recently, the production of pyroligneous acid in the pyrolysis, by collection at different temperature ranges, has been of great importance because the greatest part of its constituents are phenols and organic acids with antioxidant and antimicrobial activities.^69–72^

## 6. Pyroligneous acid

During the pyrolysis process, a crude liquid is produced from the distillation of different biomasses, which results from the thermochemical breakdown or pyrolysis of plant biomass components, such as cellulose, hemicellulose, and lignin.^73–78^ Pyroligneous acid is produced by the slow pyrolysis of plant biomass. It is a yellowish brown or dark brown liquid with an acidic pH.^79^ It is a complex highly oxygenated aqueous liquid fraction obtained by the condensation of pyrolysis vapors. Usually, the complex mixture of pyroligneous acid comprises water and different classes of organic compounds, such as guaiacols, catechols, syringols, phenols and their derivatives, benzene and its derivatives, alkyl phenyl ether, heterocyclic compounds and their derivatives, vanillins, derivatives of furans and pyrans, carboxaldehydes, hydroxyketones, hydrocarbons, organic acids, esters, ketones, carbohydrate derivatives, alkyl aryl ethers, nitrogenated derivatives, aldehydes, alcohols, acetic acid, and other carboxylic acids, in which the major compounds are organic acids and phenolics.^80,81^ Recently, there has been growing interest in the analysis of the chemical constituents of pyroligneous acid. Over 200 compounds have been found in acids obtained from different resources. Generally, for the production of pyroligneous acid, different materials or biomasses such as corn, bamboo, oak, wood, and also other agricultural by-products, have been studied comprehensively.^79^

## 7. Pyroligneous acid from walnut shells

In recent years, more and more attention has been paid to pyroligneous acid from walnut shells and, thus, the production of pyroligneous acid from the shell of the walnut fruit has been considered in some investigations. For example, the preparation of three kinds of pyroligneous acid from walnut shells over different temperature ranges (low: 90–150 °C, SP1; middle: 151–310 °C, SP2; and high: 311–550 °C, SP3) has been reported by Wei, *et al.*^72^ To establish the chemical profiles of the acids, they used the GC-MS (Gas Chromatography-Mass Spectrometry) technique and showed that the chemical constituents of the pyroligneous acids were similar: mainly phenols, organic acids, ketones, and furan derivatives. However, the contents of each constituent in the three acids varied. Also, the chemical compositions of the pyroligneous acids over the three temperature ranges were similar, but varied in their different quantities, which were mainly comprised of phenols, organic acids, ketones, and furan derivatives. Additionally, they indicated that the collection of pyroligneous acids over different temperature ranges is an effective way to pre-fractionate the chemicals contained in them and, thus, different products could be developed based on their differences in bioactivity.

In order to enrich the contents of organic acids and phenols in walnut shell pyroligneous acids, Ma, *et al.*^71^ used a pH gradient extraction method because of the differences in the acidity of these compounds. To assess the effectiveness of the extraction, they measured the contents of organic acids and phenols using an acid–base titration method and Folin colorimetric assay, respectively. By using GC-MS, they measured the chemical components of the extracts obtained using optimal concentrations of NaHCO_3_ and NaOH and reported that 5% NaHCO_3_ could enrich the highest amount of organic acids, whereas 4% NaOH could enrich the highest amount of phenols.

In a study by Zhai, *et al.*,^70^ seven kinds of pyroligneous acids were obtained over different temperature ranges (*K*_7–1_: 90 to 140 °C, *K*_7–2_: 140 to 190 °C, *K*_7–3_: 190 to 240 °C, *K*_7–4_: 240 to 290 °C, *K*_7–5_: 290 to 340 °C, *K*_7–6_: 340 to 440 °C, and *K*_7–7_: 440 to 480 °C). The results of the GC-MS analysis in this study showed that the chemical profile of pyroligneous acids varied with the pyrolysis temperature. They identified about 62 different compounds belonging to 8 groups: ketones, phenols, organic acids, esters, benzene and its derivatives, aldehydes, alcohols, and sugar derivatives.

## 8. Walnut shell pyroligneous acid constituents

### 8.1. Ketones

**Fig. 1:**
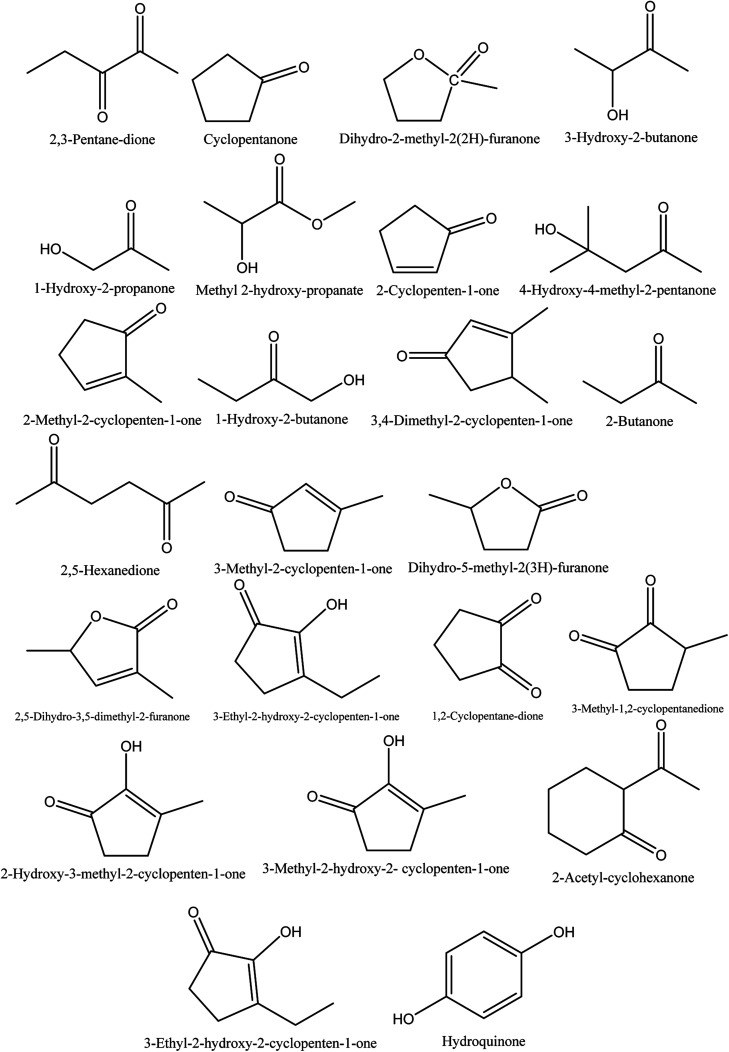
The chemical structures of isolated ketones obtained from walnut shell pyroligneous acids.

The retention time and relative contents (%) of the ketone compounds identified from walnut shell pyroligneous acids are summarized in Table 1. Collectively, 24 compounds belonging to the ketone group were reported in walnut shell pyroligneous acid and their total content is in the range 0.75–11.60%. The chemical structures of these compounds are presented in Fig. 1. Cyclopentanone, 2-cyclopenten-1-one, 2-methyl-2-cyclopenten-1-one, 1-hydroxy-2-butanone, 3-methyl-2-cyclopenten-1-one, 3-ethyl-2-hydroxy-2-cyclopenten-1-one, and hydroquinone were reported by Zhai, *et al.*,^70^ Ma, *et al.*^71^ and Wei, *et al.*^72^ Zhai, *et al.*^70^ and Ma, *et al.*^71^ identified 3-hydroxy-2-butanone and 1-hydroxy-2-propanone, and 2,5-dihydro-3,5-dimethyl-2-furanone was reported by Zhai, *et al.*^70^ and Wei, *et al.*^72^ Dihydro-2-methyl-2(2*H*)-furanone, 4-hydroxy-4-methyl-2-pentanone, 3,4-dimethyl-2-cyclopenten-1-one, dihydro-5-methyl-2(3*H*)-furanone and 3-methyl-1,2-cyclopentanedione were identified in a study by Zhai, *et al.*^70^ Ma, *et al.*^71^ found methyl 2-hydroxy-propanoate, 2-butanone, 2,5-hexanedione, 2-hydroxy-3-methyl-2-cyclopenten-1-one, and 2-acetyl-cyclohexanone. Wei, *et al.*^72^ isolated 2,3-pentane-dione, 3-ethyl-2-hydroxy-2-cyclopenten-1-one, 1,2-cyclopentane-dione, and 3-methyl-2-hydroxy-2-cyclopenten-1-one from walnut shell pyroligneous acids.

**Table 1:** Identified ketones in walnut shell pyroligneous acids

No.	Retention time (min)	Compounds	Relative content (%)	Reference
1	3.57	2,3-Pentane-dione	0.24^a^	Wei, *et al.*^72^
2	5.74	Cyclopentanone	0.18–0.23^a^	Wei, *et al.*^72^
	5.35		0.27–1.46^b^	Zhai, *et al.*^70^
	5.59		0.17^c^	Ma, *et al.*^71^
3	6.95	Dihydro-2-methyl-2(2*H*)-furanone	0.26^b^	Zhai, *et al.*^70^
4	7.38	3-Hydroxy-2-butanone	0.24^b^	Zhai, *et al.*^70^
	7.60		0.35^c^	Ma, *et al.*^71^
5	7.74	1-Hydroxy-2-propanone	0.28–1.31^b^	Zhai, *et al.*^70^
	7.94		0.87^c^	Ma, *et al.*^71^
6	8.24	Methyl 2-hydroxy-propanoate	0.20^c^	Ma, *et al.*^71^
7	9.29	2-Cyclopenten-1-one	0.43–0.95^a^	Wei, *et al.*^72^
	8.87		0.21–1.13^b^	Zhai, *et al.*^70^
	9.10		0.61–1.21^c^	Ma, *et al.*^71^
8	9.05	4-Hydroxy-4-methyl-2-pentanone	0.62^b^	Zhai, *et al.*^70^
9	9.55	2-Methyl-2-cyclopenten-1-one	0.33–0.45^a^	Wei, *et al.*^72^
	9.12		0.14–0.58^b^	Zhai, *et al.*^70^
	9.37		0.28^c^	Ma, *et al.*^71^
10	9.70	1-Hydroxy-2-butanone	0.26–0.46^a^	Wei, *et al.*^72^
	9.28		0.45–1.98^b^	Zhai, *et al.*^70^
	9.48		0.82–1.39^c^	Ma, *et al.*^71^
11	11.48	3,4-Dimethyl-2-cyclopenten-1-one	0.32^b^	Zhai, *et al.*^70^
12	11.55	2-Butanone	0.26^c^	Ma, *et al.*^71^
13	12.23	2,5-Hexanedione	0.36^c^	Ma, *et al.*^71^
14	12.69	3-Methyl-2-cyclopenten-1-one	0.29–0.60^a^	Wei, *et al.*^72^
	12.26		0.19–1.61^b^	Zhai, *et al.*^70^
	12.48		0.52^c^	Ma, *et al.*^71^
15	14.14	Dihydro-5-methyl-2(3*H*)-furanone	0.16^b^	Zhai, *et al.*^70^
16	15.35	2,5-Dihydro-3,5-dimethyl-2-furanone	0.18–0.33^a^	Wei, *et al.*^72^
	14.9		0.18–0.23^b^	Zhai, *et al.*^70^
17	15.95	3-Ethyl-2-hydroxy-2-cyclopenten-1-one	0.27–0.32^a^	Wei, *et al.*^72^
18	17.59	1,2-Cyclopentane-dione	0.27–1.38^a^	Wei, *et al.*^72^
19	18.18	3-Methyl-1,2-cyclopentanedione	0.51–2.68^b^	Zhai, *et al.*^70^
20	18.43	2-Hydroxy-3-methyl-2-cyclopenten-1-one	2.57^c^	Ma, *et al.*^71^
21	18.63	3-Methyl-2-hydroxy-2- cyclopenten-1-one	0.90–3.23^a^	Wei, *et al.*^72^
22	18.82	2-Acetyl-cyclohexanone	0.14^c^	Ma, *et al.*^71^
23	19.78	3-Ethyl-2-hydroxy-2-cyclopenten-1-one	0.33–0.91^a^	Wei, *et al.*^72^
	19.33		0.16–1.36^b^	Zhai, *et al.*^70^
	17.80		0.41^c^	Ma, *et al.*^71^
24	37.11	Hydroquinone	0.49–3.41^a^	Wei, *et al.*^72^
	36.25		0.13–1.79^b^	Zhai, *et al.*^70^
	36.75		1.90^c^	Ma, *et al.*^71^
		Total	4.46–9.14^a^	Wei, *et al.*^72^
			0.75–11.60^b^	Zhai, *et al.*^70^
			5.21–5.09^c^	Ma, *et al.*^71^

a Collected at three temperature ranges by GC-MS.

b Collected at seven temperature ranges by GC-MS.

c 5% NaHCO_3_ extract (OA_5_) and 4% NaOH extract (P_3_).

### 8.2. Organic acids

**Fig. 2:**
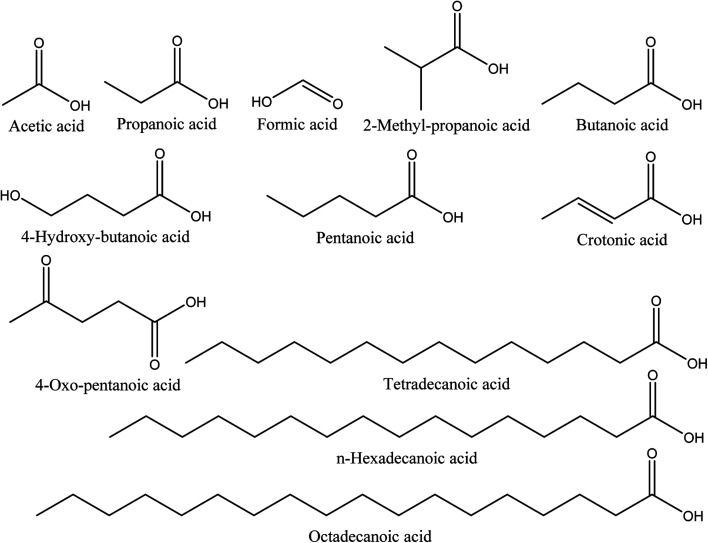
The chemical structures of organic acids identified in walnut shell pyroligneous acids.

Organic acids are the main compounds present in walnut shell pyroligneous acids. Table 2 shows the retention time, identified compounds and their relative content percentage. The chemical structures of these compounds are displayed in Fig. 2. The total content of organic acids in walnut shell pyroligneous acids is high (8.02–79.81%). Acetic acid, propanoic acid, and butanoic acid are the main organic acids.^70–72^ 2-Methyl-propanoic acid was reported by Zhai, *et al.*^70^ and Ma, *et al.*^71^ Wei, *et al.*^72^ and Ma, *et al.*^71^ detected the presence of crotonic acid. Zhai, *et al.*^70^ identified formic and pentanoic acids and three long-chain acids, tetradecanoic, *n*-hexadecanoic, and octadecanoic acid, in their investigation. Ma, *et al.*^71^ identified 4-hydroxy-butanoic and 4-oxo-pentanoic acid. Acetic acid is the dominant compound among the different organic acids reported in walnut shell pyroligneous acids.^70–72^

**Table 2:** Identified organic acids in walnut shell pyroligneous acids

No.	Retention time (min)	Compounds	Relative content (%)	Reference
1	11.25	Acetic acid	6.05–42.39^a^	Wei, *et al.*^72^
	10.73		4.59–55.81^b^	Zhai, *et al.*^70^
	10.82		10.61–63.66^c^	Ma, *et al.*^71^
2	13.11	Propanoic acid	1.31–3.03^a^	Wei, *et al.*^72^
	12.65		0.87–6.07^b^	Zhai, *et al.*^70^
	12.89		0.63–9.86^c^	Ma, *et al.*^71^
3	12.02	Formic acid	1.34^b^	Zhai, *et al.*^70^
4	13.28	2-Methyl-propanoic acid	0.49^b^	Zhai, *et al.*^70^
	13.46		1.26^c^	Ma, *et al.*^71^
5	14.90	Butanoic acid	0.40–0.76^a^	Wei, *et al.*^72^
	14.46		0.34–4.11^b^	Zhai, *et al.*^70^
	14.68		0.22^c^	Ma, *et al.*^71^
6	14.75	4-Hydroxy-butanoic acid	0.90^c^	Ma, *et al.*^71^
7	16.72	Pentanoic acid	0.21–0.55^b^	Zhai, *et al.*^70^
8	17.67	Crotonic acid	0.28^a^	Wei, *et al.*^72^
	16.18		0.23^c^	Ma, *et al.*^71^
9	26.40	4-Oxo-pentanoic acid	0.53^c^	Ma, *et al.*^71^
10	31.32	Tetradecanoic acid	1.19^b^	Zhai, *et al.*^70^
11	34.12	*n*-Hexadecanoic acid	8.41^b^	Zhai, *et al.*^70^
12	38.23	Octadecanoic acid	0.36^b^	Zhai, *et al.*^70^
		Total	8.02–45.82^a^	Wei, *et al.*^72^
			16.96–60.60^b^	Zhai, *et al.*^70^
			11.46–79.81^c^	Ma, *et al.*^71^

a Collected at three temperature ranges by GC-MS.

b Collected at seven temperature ranges by GC-MS.

c 5% NaHCO_3_ extract (OA_5_) and 4% NaOH extract (P_3_).

### 8.3. Derivatives of furan and pyran

**Fig. 3:**
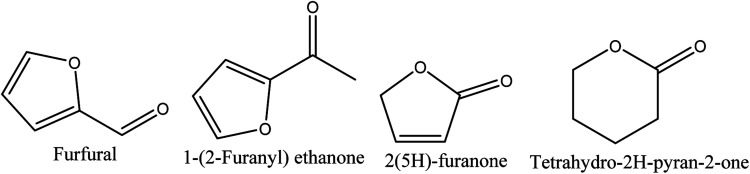
The chemical structures of the furan and pyran derivatives isolated from walnut shell pyroligneous acids.

Four different compounds belonging to furan and pyran derivatives, furfural,^70–72^ 1-(2-furanyl) ethanone,^70,72^ 2(5*H*)-furanone,^70,72^ and tetrahydro-2*H*-pyran-2-one,^71^ were reported in walnut shell pyroligneous acids (Table 3) and their structures are represented in Fig. 3. According to the obtained relative contents, furfural is the dominant compound.

**Table 3:** Identified furan and pyran derivatives in walnut shell pyroligneous acids

No.	Retention time (min)	Compounds	Relative content (%)	Reference
1	11.65	Furfural	0.61–12.06^a^	Wei, *et al.*^72^
	11.21		1.72–9.64^b^	Zhai, *et al.*^70^
	11.44		1.49–2.27^c^	Ma, *et al.*^71^
2	12.48	1-(2-Furanyl) ethanone	0.30–0.55^a^	Wei, *et al.*^72^
	12.04		0.18–0.58^b^	Zhai, *et al.*^70^
3	17.39	2(5*H*)-Furanone	0.28^a^	Wei, *et al.*^72^
	16.91		0.09–0.38^b^	Zhai, *et al.*^70^
4	18.03	Tetrahydro-2*H*-pyran-2-one	0.33^c^	Ma, *et al.*^71^
		Total	1.77–16.30^a^	Wei, *et al.*^72^
			1.49–2.60^c^	Ma, *et al.*^71^

a Collected at three temperature ranges by GC-MS.

b Collected at seven temperature ranges by GC-MS.

c 5% NaHCO_3_ extract (OA_5_) and 4% NaOH extract (P_3_).

### 8.4. Esters

**Fig. 4:**
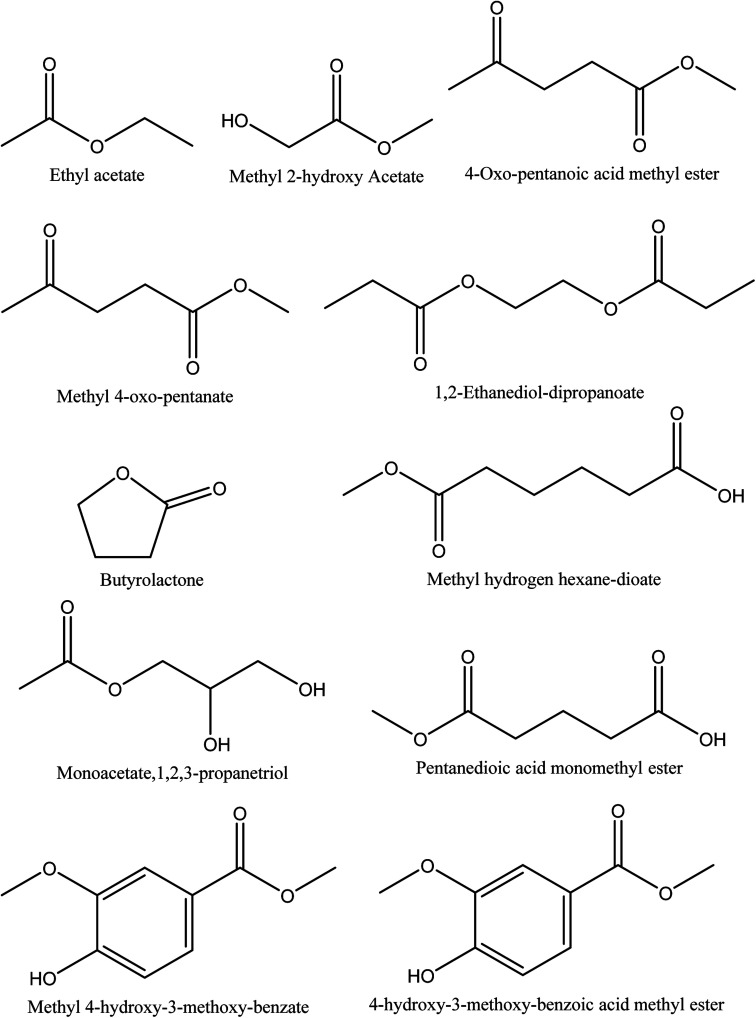
The chemical structures of ester compounds identified in walnut shell pyroligneous acids.

Eleven different ester compounds have been reported in walnut shell pyroligneous acids. The total content of this group in walnut shell pyroligneous acids is not high (Table 4). The chemical structures of isolated ester compounds in walnut shell pyroligneous acids are depicted in Fig. 4. Ethyl acetate, 4-oxo-pentanoic acid methyl ester, pentanedioic acid monomethyl ester, 1,2-ethanediol-dipropanoate, and 4-hydroxy-3-methoxy-benzoic acid methyl ester were isolated by Zhai, *et al.*^70^ Ma, *et al.*^71^ reported methyl 2-hydroxy acetate and monoacetate,1,2,3-propanetriol. Wei, *et al.*^72^ detected methyl 4-oxo-pentanate, methyl hydrogen hexane-dioate, and methyl 4-hydroxy-3-methoxy-benzate. Butyrolactone was also isolated by Wei, *et al.*^72^ and Zhai, *et al.*^70^

**Table 4:** Identified esters in walnut shell pyroligneous acids

No.	Retention time (min)	Compounds	Relative content (%)	Reference
1	5.67	Ethyl acetate	0.22–0.42^b^	Zhai, *et al.*^70^
2	9.72	Methyl 2-hydroxy acetate	0.19^c^	Ma, *et al.*^71^
3	13.34	4-Oxo-pentanoic acid methyl ester	0.11–0.35^b^	Zhai, *et al.*^70^
4	13.77	Methyl 4-oxo-pentanate	0.29–0.34^a^	Wei, *et al.*^72^
5	13.88	1,2-Ethanediol-dipropanoate	0.09^b^	Zhai, *et al.*^70^
6	14.98	Butyrolactone	0.22–0.82^a^	Wei, *et al.*^72^
	14.51		0.31^b^	Zhai, *et al.*^70^
7	19.61	Methyl hydrogen hexane-dioate	0.30^a^	Wei, *et al.*^72^
8	25.00	Monoacetate,1,2,3-propanetriol	0.16^c^	Ma, *et al.*^71^
9	27.74	Pentanedioic acid monomethyl ester	0.40^b^	Zhai, *et al.*^70^
10	30.52	Methyl 4-hydroxy-3-methoxy-benzate	0.20–0.25^a^	Wei, *et al.*^72^
11	30.06	4-Hydroxy-3-methoxy-benzioc acid methyl ester	0.21^b^	Zhai, *et al.*^70^
		Total	0.42–1.71^a^	Wei, *et al.*^72^
			0.22–0.92^b^	Zhai, *et al.*^70^
			0.35^c^	Ma, *et al.*^71^

a Collected at three temperature ranges by GC-MS.

b Collected at seven temperature ranges by GC-MS.

c 5% NaHCO_3_ extract (OA_5_) and 4% NaOH extract (P_3_).

### 8.5. Phenolics

**Fig. 5:**
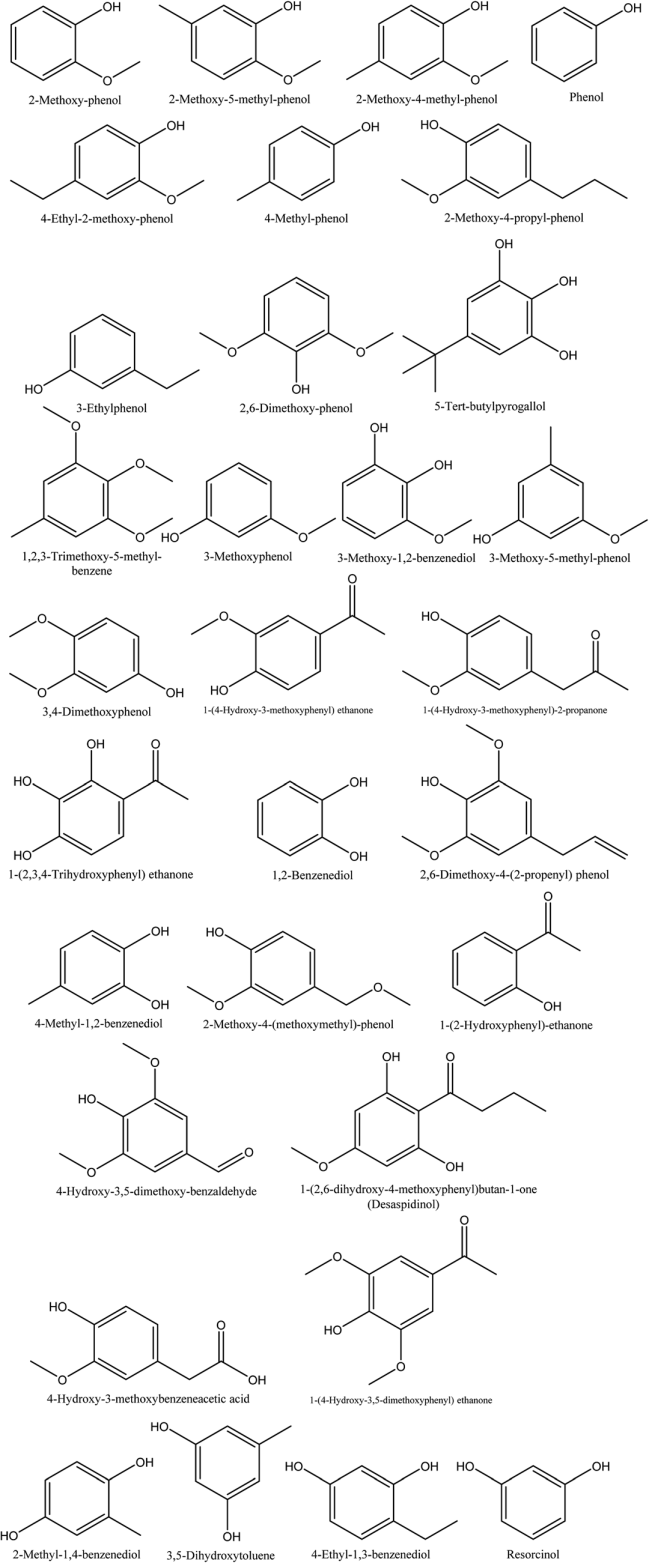
The chemical structures of phenol and its derivatives in walnut shell pyroligneous acids.

The smoky odor of pyroligneous acid is due to the presence of phenolic compounds, namely guaiacol, alkyl guaiacols, syringol, and alkyl syringols.^79^ Investigations performed on walnut shell pyroligneous acid show that phenol and its derivatives are the main constituents, and various compounds belonging to this group have been isolated, as shown in Table 5 and Fig. 5. In total, 32 different phenolic compounds have been reported in walnut shell pyroligneous acid. 2-Methoxy-phenol, 2-methoxy-5-methyl-phenol, phenol, 4-ethyl-2-methoxy-phenol, 4-methyl-phenol, 2,6-dimethoxy-phenol, 3-methoxy-1,2-benzenediol, 1-(4-hydroxy-3-methoxyphenyl)-2-propanone, 1,2-benzenediol, 4-hydroxy-3,5-dimethoxy-benzaldehyde, and 1-(4-hydroxy-3,5-dimethoxyphenyl) ethanone are the main phenolic compounds.^70–72^ Other phenolics, such as 2-methoxy-4-methyl-phenol,^70,71^ 2-methoxy-4-propyl-phenol, 2,6-dimethoxy-4-(2-propenyl) phenol, 2-methyl-1,4-benzenediol,^70,72^ 1-(4-hydroxy-3-methoxyphenyl) ethanone, 4-methyl-1,2-benzenediol,^71,72^ 3-ethylphenol, 3-methoxyphenol, 3,4-dimethoxyphenol, 1-(2,3,4-trihydroxyphenyl) ethanone, 4-hydroxy-3-methoxybenzeneacetic acid, 3,5-dihydroxytoluene, 4-ethyl-1,3-benzenediol, resorcinol,^72^ 5-*tert*-butylpyrogallol, 3-methoxy-5-methyl-phenol, 2-methoxy-4-(methoxymethyl)-phenol, desaspidinol,^70^ 1,2,3-trimethoxy-5-methyl-benzene, 3-methoxy-1,2-benzenediol, 1-(2-hydroxyphenyl)-ethanone,^71^ have been also identified in walnut shell pyroligneous acid.

**Table 5:** Identified phenol and derivatives in walnut shell pyroligneous acids

No.	Retention time (min)	Compounds	Relative content (%)	Reference
1	19.24	2-Methoxy-phenol	4.48–6.69^a^	Wei, *et al.*^72^
	18.77		0.28–9.10^b^	Zhai, *et al.*^70^
	19.02		9.76–0.58^c^	Ma, *et al.*^71^
2	20.89	2-Methoxy-5-methyl-phenol	1.77–3.13^a^	Wei, *et al.*^72^
	20.19		0.24–0.42^b^	Zhai, *et al.*^70^
	20.44		3.22^c^	Ma, *et al.*^71^
3	20.42	2-Methoxy-4-methyl-phenol	0.72–4.27^b^	Zhai, *et al.*^70^
	20.68		2.82–0.19^c^	Ma, *et al.*^71^
4	21.73	Phenol	1.62–5.27^a^	Wei, *et al.*^72^
	21.24		1.63–23.78^b^	Zhai, *et al.*^70^
	21.51		2.24–0.20^c^	Ma, *et al.*^71^
5	22.11	4-Ethyl-2-methoxy-phenol	0.70–1.74^a^	Wei, *et al.*^72^
	21.64		0.24–3.20^b^	Zhai, *et al.*^70^
	21.90		1.28^c^	Ma, *et al.*^71^
6	22.96	4-Methyl-phenol	0.64–1.15^a^	Wei, *et al.*^72^
	22.49		0.20–4.18^b^	Zhai, *et al.*^70^
	22.75		6.81^c^	Ma, *et al.*^71^
7	23.37	2-Methoxy-4-propyl-phenol	0.25^a^	Wei, *et al.*^72^
	22.90		0.18–6.26^b^	Zhai, *et al.*^70^
8	24.53	3-Ethylphenol	0.22^a^	Wei, *et al.*^72^
9	25.79	2,6-Dimethoxy-phenol	8.38–13.80^a^	Wei, *et al.*^72^
	25.32		3.00–18.30^b^	Zhai, *et al.*^70^
	25.60		17.28–3.71^c^	Ma, *et al.*^71^
10	27.38	5-Tert-butylpyrogallol	0.96–4.75^b^	Zhai, *et al.*^70^
11	27.65	1,2,3-Trimethoxy-5-methyl-benzene	1.41 0.17^c^	Ma, *et al.*^71^
12	28.37	3-Methoxyphenol	0.24^a^	Wei, *et al.*^72^
13	29.24	3-Methoxy-1,2-benzenediol	1.48–6.62^a^	Wei, *et al.*^72^
	28.83		0.22^b^	Zhai, *et al.*^70^
	28.62		0.66–0.19^c^	Ma, *et al.*^71^
14	28.98	3-Methoxy-5-methyl-phenol	0.2^b^	Zhai, *et al.*^70^
15	29.03	3-Methoxy-1,2-benzenediol	5.35–0.33^c^	Ma, *et al.*^71^
16	30.33	3,4-Dimethoxyphenol	0.86–2.17^a^	Wei, *et al.*^72^
17	30.97	1-(4-Hydroxy-3-methoxyphenyl) ethanone	0.31–0.47^a^	Wei, *et al.*^72^
	30.78		0.64^c^	Ma, *et al.*^71^
18	31.21	1-(4-Hydroxy-3-methoxyphenyl)-2-propanone	1.56–2.63^a^	Wei, *et al.*^72^
	30.73		1.10–1.49^b^	Zhai, *et al.*^70^
	31.02		1.91–0.41^c^	Ma, *et al.*^71^
19	31.32	1-(2,3,4-Trihydroxyphenyl) ethanone	0.28^a^	Wei, *et al.*^72^
20	31.82	1,2-Benzenediol	1.45–13.48^a^	Wei, *et al.*^72^
	31.44		8.77^b^	Zhai, *et al.*^70^
	31.60		8.78^c^	Ma, *et al.*^71^
21	32.15	2,6-Dimethoxy-4-(2-propenyl) phenol	0.22–0.82^a^	Wei, *et al.*^72^
	29.19		0.41–1.65^b^	Zhai, *et al.*^70^
22	32.72	4-Methyl-1,2-benzenediol	0.65–5.03^a^	Wei, *et al.*^72^
	32.50		3.07^c^	Ma, *et al.*^71^
23	33.25	2-Methoxy-4-(methoxymethyl)-phenol	0.24^b^	Zhai, *et al.*^70^
24	33.96	1-(2-Hydroxyphenyl)-ethanone	0.35^c^	Ma, *et al.*^71^
25	35.29	4-Hydroxy-3,5-dimethoxy-benzaldehyde	0.24–0.28^a^	Wei, *et al.*^72^
	34.68		0.25–0.50^b^	Zhai, *et al.*^70^
	35.02		0.28^c^	Ma, *et al.*^71^
26	35.91	Desaspidinol	0.9–1.68^b^	Zhai, *et al.*^70^
27	35.97	4-Hydroxy-3-methoxybenzeneacetic acid	0.44^a^	Wei, *et al.*^72^
28	36.25	1-(4-Hydroxy-3,5-dimethoxyphenyl) ethanone	0.42–0.78^a^	Wei, *et al.*^72^
	35.55		0.45–0.56^b^	Zhai, *et al.*^70^
	35.96		0.96^c^	Ma, *et al.*^71^
29	36.51	2-Methyl-1,4-benzenediol	0.44–1.92^a^	Wei, *et al.*^72^
	35.67		0.49–1.48^b^	Zhai, *et al.*^70^
30	37.72	3,5-Dihydroxytoluene	0.23^a^	Wei, *et al.*^72^
31	37.83	4-Ethyl-1,3-benzenediol	0.28^a^	Wei, *et al.*^72^
32	38.97	Resorcinol	0.38^a^	Wei, *et al.*^72^
		Total	29.00–62.86^a^	Wei, *et al.*^72^
			3.18–55.27^b^	Zhai, *et al.*^70^

a Collected at three temperature ranges by GC-MS.

b Collected at seven temperature ranges by GC-MS.

c 5% NaHCO_3_ extract (OA_5_) and 4% NaOH extract (P_3_).

### 8.6. Alcohols

**Fig. 6:**
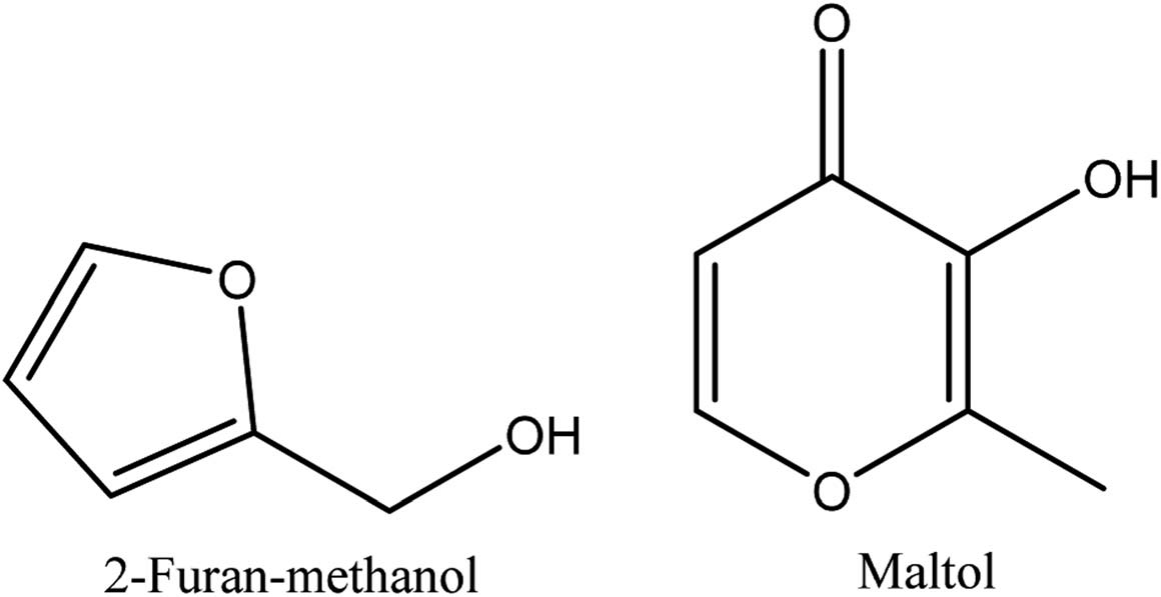
The chemical structures of alcohols identified in walnut shell pyroligneous acids.

2-furan-methanol and maltol are the alcohols identified in walnut shell pyroligneous acids.^70–72^ Their total contents are not significant, as shown in Table 6. Fig. 6 illustrates the chemical structures of the compounds related to alcohols.

**Table 6:** Identified alcohols in walnut shell pyroligneous acids

No.	Retention time (min)	Compounds	Relative content (%)	Reference
1	15.56	2-Furan-methanol	0.78–3.15^a^	Wei, *et al.*^72^
	15.15		0.33–2.30^b^	Zhai, *et al.*^70^
2	21.00	Maltol	0.43–0.55^a^	Wei, *et al.*^72^
	20.80		1.40–0.42^c^	Ma, *et al.*^71^
		Total	0.43–0.55^a^	Wei, *et al.*^72^
			0.33–2.30^b^	Zhai, *et al.*^70^

a Collected at three temperature ranges by GC-MS.

b Collected at seven temperature ranges by GC-MS.

c 5% NaHCO_3_ extract (OA_5_) and 4% NaOH extract (P_3_).

### 8.7. Aldehydes

**Fig. 7:**
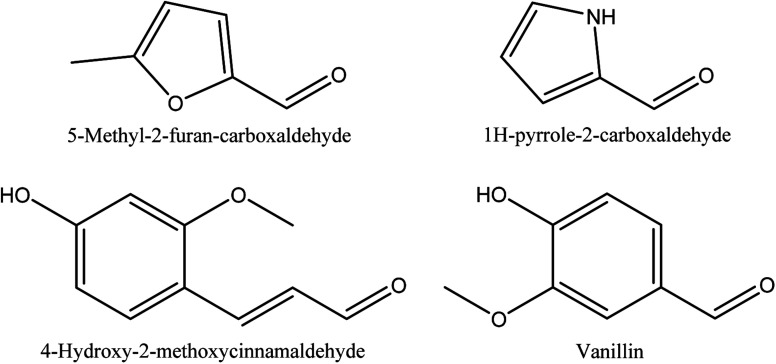
The chemical structures of aldehyde compounds identified in walnut shell pyroligneous acids.

Table 7 and Fig. 7 show the retention time, content and chemical structures of 5-methyl-2-furan-carboxaldehyde, 1*H*-pyrrole-2-carboxaldehyde, vanillin, and 4-hydroxy-2-methoxycinnamaldehyde as isolated aldehydes from walnut shell pyroligneous acids.^70,72^

**Table 7:** Identified aldehydes in walnut shell pyroligneous acids

No.	Retention time (min)	Compounds	Relative content (%)	Reference
1	13.88	5-Methyl-2-furan-carboxaldehyde	0.26–0.41^a^	Wei, *et al.*^72^
	13.44		0.20–0.51^b^	Zhai, *et al.*^70^
2	21.56	1*H*-Pyrrole-2-carboxaldehyde	0.12–0.31^b^	Zhai, *et al.*^70^
3	29.99	Vanillin	0.28–4.17 a	Wei, *et al.*^72^
4	38.84	4-Hydroxy-2-methoxycinnamaldehyde	0.41^b^	Zhai, *et al.*^70^
		Total	0.28–4.17^a^	Wei, *et al.*^72^
			0.25–11.74^b^	Zhai, *et al.*^70^

a Collected at three temperature ranges by GC-MS.

b Collected at seven temperature ranges by GC-MS.

### 8.8. Alkyl aryl ether and benzene derivatives

**Fig. 8:**
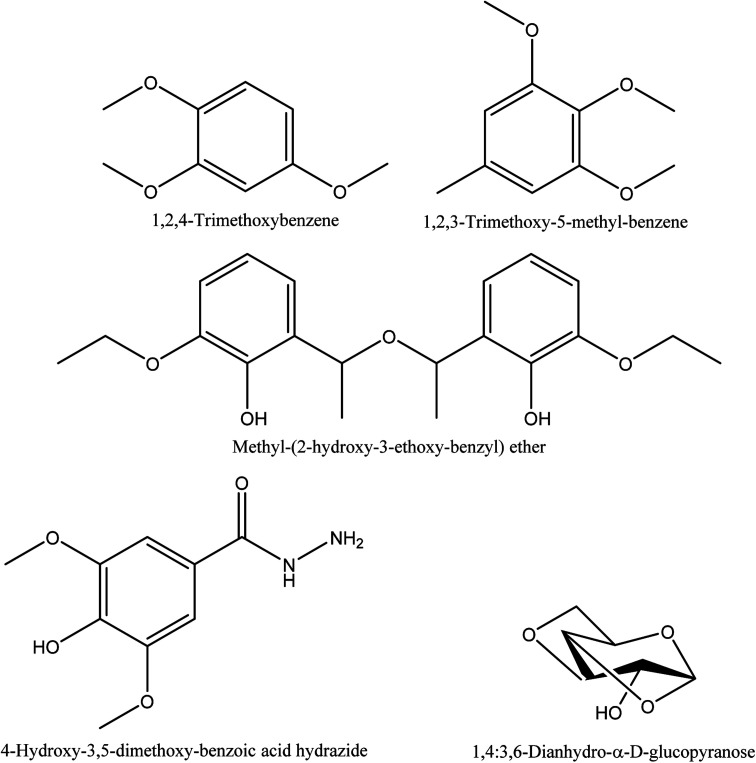
The chemical structures of alkyl aryl ether, benzene and sugar derivatives identified in walnut shell pyroligneous acids.

1,2,4-trimethoxybenzene, 1,2,3-trimethoxy-5-methyl-benzene, methyl-(2-hydroxy-3-ethoxy-benzyl) ether, and 4-hydroxy-3,5-dimethoxy-benzoic acid hydrazide are the characterized alkyl aryl ether and benzene derivations from walnut shell pyroligneous acids^70,72^ (see Table 8 and Fig. 8 for more details).

**Table 8:** Identified alkyl aryl ether and benzene derivatives in walnut shell pyroligneous acids

No.	Retention time (min)	Compounds	Relative content (%)	Reference
1	27.02	1,2,4-Trimethoxybenzene	1.48–5.83 a	Wei, *et al.*^72^
	26.56		0.61–8.02^b^	Zhai, *et al.*^70^
2	27.83	1,2,3-Trimethoxy-5-methyl-benzene	1.68–3.07^a^	Wei, *et al.*^72^
	21.96		0.19^b^	Zhai, *et al.*^70^
3	35.43	Methyl-(2-hydroxy-3-ethoxy-benzyl) ether	0.23–0.24^b^	Zhai, *et al.*^70^
4	35.08	4-Hydroxy-3,5-dimethoxy-benzoic acid hydrazide	0.21–0.37^b^	Zhai, *et al.*^70^
		Total	3.56–8.90 a	Wei, *et al.*^72^
			021–8.68^b^	Zhai, *et al.*^70^

a Collected at three temperature ranges by GC-MS.

b Collected at seven temperature ranges by GC-MS.

### 8.9. Sugar derivatives

1,4:3,6-Dianhydro-α-d-glucopyranose, a sugar derivative, has been shown by Zhai, *et al.*^70^ to occur in walnut shell pyroligneous acid (refer to Table 9 and Fig. 8).

**Table 9:** Identified sugar derivatives in walnut shell pyroligneous acids

No.	Retention time (min)	Compounds	Relative content (%)	Reference
1	27.25	1,4:3,6-Dianhydro-α-d-glucopyranose	0.32–0.77^a^	Zhai, *et al.*^70^
		Total	0.32–0.77^a^	Zhai, *et al.*^70^

a Collected at seven temperature ranges by GC-MS.

## 9. Functional applications of pyroligneous acid

Pyroligneous acid has been widely used in diverse areas, as an antioxidant in the food industry, as antimicrobials and anti-inflammatories, as a plant growth stimulator in agriculture, as a coagulant for natural rubber, and as termiticidal and pesticidal agents in medicine. It is a source of valuable chemicals and it imparts a smoky flavor to food.^79^ It has been reported that the anti-inflammatory effect of oak wood vinegar inactivates STAT3, a signal transducer and activator of transcription factor 3, in DNCB-induced dermatitis in mice models, and that epithelial proliferation is inhibited by oak wood vinegar.^82^ In agriculture, the rooting and germination properties of seeds could be promoted by pyroligneous acid.^83^ In an investigation by Chen, *et al.*,^84^ pyroligneous acid was added into pig manure compost to reduce total nitrogen loss and to control the mobility of Zn and Cu and it is safe for use as an animal feed additive.^85^ Baimark and Niamsa^86^ obtained pyroligneous acid from bamboo and coconut shell and then used it as an antifungal and coagulator agent to produce natural rubber sheets due to its acidic properties. Wendin, *et al.*^87^ showed that pyroligneous acid imparts much-appreciated organoleptic properties to smoked food. Pyroligneous acid from bamboo demonstrated superoxide anion scavenging activity and antioxidant activity.^88^ Loo, *et al.*^89^ reported antioxidant and free radical scavenging activities of pyroligneous acid from a mangrove plant, *Rhizophora apiculata*, and found that concentrated pyroligneous acid extract showed superior free radical scavenging activity. All these results suggest that pyroligneous acid has the potential to be considered a resource of natural antioxidants. Different valuable compounds, such as syringol, catechol, acetol, levoglucosan, ketones, alcohols, and acids, can also be obtained from pyroligneous acid.^89–92^ Additionally, it has been reported that pyroligneous acid can be used as a sterilizing agent, deodorizer, and fertilizer.^89,93^ The antioxidant and antimicrobial activities of pyroligneous acids are their most important feature and, thus, have received much interest over recent years. It is well known that the strong biological activities of pyroligneous acids can be related to their high concentrations of phenolic compounds and organic acids.^72,94^

## 10. Uses of walnut shell pyroligneous acid

### 10.1. Antioxidant activity

Research has demonstrated that phenolic compounds can be used as reductants and antioxidants because they exhibit strong free radical scavenging activities, reducing power, and high antioxidant capability. Recently, more consideration has been paid to the antioxidant activity of phenolic compounds originating from plants, since synthetic antioxidants show some side effects, and thus there is a tendency to substitute synthetic antioxidants with natural plant-derived antioxidants. In this regard, pyroligneous acid can be considered as a source for obtaining antioxidants because it is rich in phenolic compounds, which are pyrolytic products of lignin and hemicellulose, comprising 30–60% of the total organic compounds in the acid, and can be used as a source of food antioxidants.^79^

Walnut shell pyroligneous acids were also obtained at different temperatures and their antioxidant capacities studied for use as antioxidants. In this way, Wei, *et al.*^72^ investigated the antioxidant activities of walnut shell pyroligneous acids from the aspects of DPPH free radical scavenging capacity, hydroxyl free radical scavenging capacity, superoxide anion radical scavenging capacity, reducing power, and anti-lipid peroxidation capacity. The authors found that all the pyroligneous acids exhibited antioxidant activity. The antioxidant activity exhibited a significant dosage-dependence effect, and SP3 collected from the high-temperature range showed the strongest antioxidant activity, followed by those from the middle (SP2) and low (SP1) temperature ranges. Their results indicated that all the pyroligneous acids exhibited antioxidant activity, but at different levels. They reported that the strongest antioxidant activity of SP3 was due to its highest content of phenols among the three acids. Finally, the authors claimed that SP3 can hopefully be developed as a food antioxidant due to its excellent performance in anti-lipid peroxidation.

According to Ma, *et al.*'s^71^ results, the extracts obtained with different concentrations of NaOH show different antioxidant activities, which increased along with an increase in phenol content. They suggested that the extract obtained with 4% NaOH showed the best antioxidant activity. With a concentration of 0.01 mg ml^−1^, the rate of DPPH free radical scavenging capability was 95.21%, the ferric reducing power was 51.3 mg L^−1^ (FeSO_4_ equivalent), the reducing power to molybdenum salt was 1.936 mg mg^−1^ (ascorbic acid equivalent), and the inhibition rate of oleic acid oxidation was 98.02%. The authors found that basic solution extraction was an excellent method for enriching organic acids and phenols. They concluded that the extracts had potential for development as antioxidant agents because they exhibit strong antioxidant activities.

### 10.2. Antimicrobial activity

It has been shown that the strong antimicrobial activity of pyroligneous acid is related to its high content of organic acids and phenolic compounds.^71,72,79^ In an investigation, Wei, *et al.*^69^ tried to evaluate the antimicrobial activities of walnut shell pyroligneous acids. They collected pyroligneous acids over three different temperature ranges (190 to 150, 150 to 310, and 310 to 550 °C). The results of this study showed that the antimicrobial activities of pyroligneous acids at 150 to 310, and 310 to 550 °C were higher than those of pyroligneous acids at 190 to 150 °C.

In another investigation, the antimicrobial activities of the acids from three temperature ranges were tested, and the chemical constituents of one of the three acids that demonstrated the strongest antimicrobial activity were analyzed by GC-MS. It was found that pyroligneous acids that were collected from different temperature ranges exhibited different antimicrobial activities, indicating that collecting pyroligneous acids from different temperature ranges is an effective method for the pre-fractionation of the acids. To study the antimicrobial and antifungal activities of walnut shell pyroligneous acid extracted with NaHCO_3_ at different concentrations, four bacteria (*Staphylococcus aureus*, *Bacillus subtilis*, *Escherichia coli*, and *Bacterium prodigious*) and four fungi (*Alternaria solani*, *Verticillium dahliae*, *Glomerella cingulata*, and *Botrytis cinerea*) were used. The extracts were prepared into solutions with a content of 20 mg ml^−1^. The authors reported that the extracts obtained with different concentrations of NaHCO_3_ showed different antimicrobial activities and they showed dosage dependency. The extract obtained with 5% NaHCO_3_ exhibited the best antimicrobial activity. They observed that at a concentration of 5 mg ml^−1^, the inhibition rates to tested bacteria were 85.31–90.26%, and 75–78.17% to fungi tested at a concentration of 20 mg ml^−1^. Their results for the antimicrobial activity of the organic acids and antioxidant activity of the phenols showed that the enriched organic acids exhibited high antimicrobial activity, and the enriched phenols exhibited antioxidant activity at low concentrations and demonstrated dosage dependency. The authors established that basic solution extraction was an excellent method of enriching organic acids and phenols. In conclusion, they claimed that the extracts could potentially be considered as antimicrobial agents due to their strong antimicrobial activities.^71^

### 10.3. Fertilizing agent

Zhai, *et al.*^70^ investigated the biological activities of walnut shell pyroligneous acids. To this end, they used the pyroligneous acid in a foliar spray for rape (*Brassica campestris* L.). They found that walnut shell pyroligneous acid at a low concentration significantly enhanced the content of soluble protein and the activity of SOD (superoxide dismutase) in the rape leaves.

## 11. Conclusions

Walnut shell pyrolysis can be considered an effective method of management of this agricultural waste product, because it provides a route for the production of pyroligneous acid with antioxidant, antimicrobial, and plant growth stimulating properties, and a source for various value-added compounds. In addition, the utilization of walnut shells could help to reduce the environmental pollution resulting from its being discarded or burnt. Thus, a study of the utilization of walnut shells as an important bioresource for pyroligneous acid production with different uses has been paid much interest over recent years, but the investigations performed reporting walnut shell pyroligneous acid are not adequate for their wide application in industry and thus additional studies will be required in the future.

## Conflicts of interest

There are no conflicts to declare.

## Supplementary Material
